# Multivariate Streamflow Simulation Using Hybrid Deep Learning Models

**DOI:** 10.1155/2021/5172658

**Published:** 2021-10-27

**Authors:** Eyob Betru Wegayehu, Fiseha Behulu Muluneh

**Affiliations:** School of Civil and Environmental Engineering, Addis Ababa Institute of Technology, Addis Ababa University, Addis Ababa, Ethiopia

## Abstract

Reliable and accurate streamflow simulation has a vital role in water resource development, mainly in agriculture, environment, domestic water supply, hydropower generation, flood control, and early warning systems. In this context, these days, deep learning algorithms have got enormous attention due to their high-performance simulation capacity. In this study, we compared multilayer perceptron (MLP), long short-term memory (LSTM), and gated recurrent unit (GRU) with the proposed new hybrid models, including CNN-LSTM and CNN-GRU. Hence, we can simulate one-step daily streamflow in different agroclimatic conditions, rolling time windows, and a range of variable input combinations. The analysis used daily multivariate and multisite time series data collected from Awash River Basin (Borkena watershed: Ethiopia) and Tiber River Basin (Upper Tiber River Basin: Italy) stations. The datasets were subjected to rigorous quality control processes. Consequently, it rolled to a different time lag to remove noise in the time series and further split into training and testing datasets using a ratio of 80 : 20, respectively. Finally, the results showed that integrating the GRU layer with the convolutional layer and using monthly rolled average daily input time series could substantially improve the simulation of streamflow time series.

## 1. Introduction

One of the emerging research areas in hydrology is hydrological simulation [1], through which catchment responses are evaluated in terms of meteorological forcing variables. Hydrological simulation is also crucial for water resource planning and management, such as flood prevention, water supply distribution, hydraulic structure design, and reservoir operation [2, 3]. However, river flow simulation is not an easy task since river flow time series are commonly random, dynamic, and chaotic. The relationship between streamflow generation and other hydrologic processes is nonlinear, which is controlled not only by external climatic factors and global warming but also by physical catchment characteristics.

Stream flows are mostly recorded at river gauging stations. However, different research studies show that the availability of gauging station records is generally decreasing in most parts of the world [4]. Tourian et al. [5] gathered a time series plot of the number of stations with available discharge data from the Global Runoff Data Centre (GRDC). This time series indicates a decline in the total monitored annual stream flows between 1970 and 2010. Besides, inadequate discharge observation and malfunctioned gauging stations worsen the situation in developing countries [6]. Sparsely distributed rain gauge stations in Ethiopia also limit the performance of physical hydrological models. Therefore, research studies on the robustness of innovative discharge data estimation models are undeniably important.

Streamflow simulation models in the literature generally are divided into two: (1) process or physical-based models that are generated from catchment characteristics and (2) data-driven models that depend on historically collected data [2, 3, 7]. Process-based models commonly use the experimental formula that provides insight into physical characteristics and has extensive data requirements. On the other hand, data-driven models are suitable and can function easily without considering the internal physical mechanism of the watershed system [2, 3, 7].

Artificial neural networks (ANNs) are the most used and studied “black-box” models. They are utilized in many scientific and technological areas than the list of available black-box algorithms, such as support vector machine (SVM), genetic programming (GP), fuzzy logic (FL), recurrent neural network (RNN), and long short-term memory (LSTM) [7, 8]. ANN is available in different functionalities and architectural forms, from simple to advanced levels. A recurrent neural network (RNN) is one of the advanced ANN architectures. It has been considered a specially designed deep learning network for time series analysis that quickly adapts to temporal dynamics using previous time step information [2]. However, RNN cannot capture long-time dependencies, and it is susceptible to vanishing and exploding gradients.

Couta et al. suggested advanced RNN or long short-term memory (LSTM) as one of the most effective approaches [8]. The LSTM unit has a cell that comprises an input gate, an output gate, and a forget gate [9]. Due to these gates, the LSTM model has shown promising results in different applications, including speech recognition, time series modelling, natural language processing, handwriting recognition, and traffic flow simulation [3, 10]. Studies have also shown that LSTM has powerful performance for streamflow simulation over different powerful multilayered (ML) tools [3, 11]. Campos et al. [10] applied autoregressive integrated moving average (ARIMA) and LSTM network to forecast floods on four Para' iba do Sul's River stations in Brazil. Aljahdali et al. [7] also compared the LSTM network and layered RNN to forecast streamflow in the USA's two rivers, the Black and Gila rivers. A recent article by Rahimzad et al. [12] used time-lagged Qt-1, Qt-2, and other climatic variables to forecast Qt in the future and concluded that the LSTM network outperforms linear regression (LR), multilayer perceptron (MLP), and support vector machine (SVM) in forecasting daily streamflow.

A few years back, Cho et al. [13] introduced gated recurrent units (GRUs) similar to LSTM with a forget gate which have fewer parameters than LSTM, as it lacks an output gate. GRU's capacities in speech signal modelling and natural language processing were similar to those of LSTM. However, there are debates on the relative performance of these two architectures for streamflow and reservoir inflow simulation, which is not well studied with different timescales and environments.

Notwithstanding the difference in their performance, selecting appropriate time series models from various known deep learning network architectures is difficult. LSTMs and GRUs are not always the ideal sequence prediction option. However, simulation with better prediction accuracy, fast running time, and less complicated models requires more research. Hence, this comparative analysis on the network architectures helps decide the optimized alternative for time series analysis. Recently, different hybrid deep learning models are getting wide attention from researchers in various fields of study. Chen et al. [14] used convolutional neural network (CNN), LSTM, and hybrid CNN-LSTM models for nitrogen oxide emission prediction. They concluded that CNN-LSTM has an accurate and stable forecast of periodic nitrogen oxide emissions from the refining industry. Moreover, Li et al. [15] used univariate and multivariate time series data as input for LSTM and CNN-LSTM models. Hence, for the analysis of air quality using particulate matter (PM2.5) concentration prediction, the proposed multivariate CNN-LSTM model gives the best result due to low error and short training time.

The integration of CNN and LSTM models benefits time series prediction models such that the LSTM model can efficiently capture long time sequences of pattern information. In contrast, CNN models can filter out the noise of the input data and extract more valuable features, which could increase the accuracy of the prediction model [16]. Moreover, integrating CNN with GRU can also lead us to robust preprocessing of data, providing a viable option to improve the model's accuracy [17]. Even though combining CNN with LSTM showed remarkable results in different studies, its application in hydrological fields still demands more research [18]. Muhammad et al. [19] used LSTM, GRU, and hybrid CNN-GRU models for streamflow simulation based on 35 years of Model Parameter Estimation Experiment (MOPEX) dataset of 10 river basins in the USA. They revealed that the proposed hybrid model outperforms the conventional LSTM; nevertheless, the performance is almost the same with GRU. Recently, Barzegar et al. [20] studied short-term water quality variable prediction using a hybrid CNN-LSTM model and effectively captured low and high water quality variables, mainly dissolved oxygen concentrations.

Screening input variables for different model architectures is also a challenging task for the researchers. Even though rainfall, evaporation, and temperature are causal variables for streamflow modelling, data availability and study objectives limit the choice variability [21]. Van et al. [21] discussed that applying temperature and evapotranspiration input nodes into the model increases the network complexity and causes overfitting. In contrast, Parisouj et al. [22] concluded that using readily available input variables such as temperature and precipitation for data-driven streamflow simulation will provide a reliable result. Hence, this research will contribute a step to this debate by testing different input combinations of various climatic regions in the performance of the proposed models.

To the best of our knowledge, we identify minimal literature that shows the performance variation of different hybrid models for streamflow simulation in various input variability conditions at once. Thus, we compared various forms of hybrid CNN-LSTM and CNN-GRU architectures with the classical MLP, GRU, and LSTM networks to simulate single-step streamflow using two climatic regions, available precipitation, and minimum and maximum temperature data. Moreover, the study tests the hybrid models with different layer arrangements and applies Keras tuner to optimize model hyperparameters. In general, the primary objective of this study will be to test the performance variation of the proposed models with extreme input variability conditions, which includes climatic, input combination, input time window, and average rolling time window variability.

This study used different open-source software and machine learning libraries, including Python 3.6 for programming, NumPy, pandas, Scikit-learn, Hydroeval, Statsmodels, and Matplotlib libraries. All were used for data preprocessing, evaluation, and graphical interpretation. Moreover, TensorFlow and Keras deep learning frameworks were employed for modelling deep learning architectures.

## 2. Study Area

**Figure 1:**
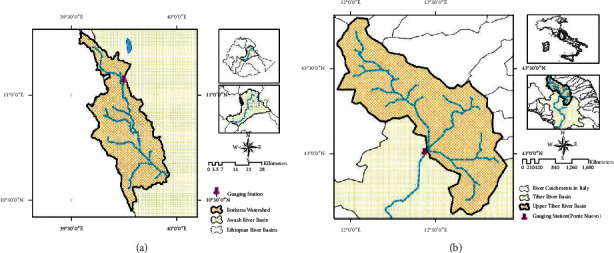
Location of case study areas. (a) Borkena. (b) UTRB.

In the present study, two river subcatchments were selected in two climatic regions: the Awash River Basin, Borkena subcatchment in Ethiopia (Figure 1(a)), and the Upper Tiber River Basin in Italy (Figure 1(b)).

### 2.1. Borkena Watershed (Ethiopia)

The first case study area is in the Borkena watershed at the Kombolcha station outlet, located in the upper part of the Awash River Basin in the northern part of Ethiopia. The mainstream of the watershed emanates from Tosa mountain, which is found near Dessie town. The area's altitude ranges from 1,775 m at the lowest site near Kombolcha to 2,638 m at the highest site upstream of Dessie. The main rainy season of this watershed is from July to September.

### 2.2. Upper Tiber River Basin (Italy)

The second case study area is located in the Upper Tiber River Basin (UTRB) in Italy. The Tiber River Basin (TRB) is the second-largest catchment in Italy [23]. Geographically, the basin is located between 40.5°N to 43°N latitudes and 10.5° E to 13° E longitudes, covering about 17,500 km^2^ that occupies roughly 5% of the Italian territory. The Upper Tiber River Basin (UTRB) is part of the TRB, covering 4145 km2 (∼20% of the TRB) with its outlet at Ponte Nuovo. The elevation of the catchment ranges from 148 to 1561 m above sea level. The area's climate is the Mediterranean, with precipitation mainly occurring from autumn (March to May) to spring (September to November). The intense rainfall highly influences the basin's hydrology at the upstream part that causes frequent floods in the downstream areas [24].

## 3. Data Source and Preprocessing

Borkena's required hydrological and metrological datasets were collected from the Ministry of Water Irrigation and Energy (MoWIE) of Ethiopia and the National Meteorological Agency of Ethiopia (NMA), respectively. UTRB's datasets were collected from the National Research Council of Italy (CNR) and archived for public use with the Water Resource Management and Evaluation (WRME) platform in the following link: http://hydrogate.unipg.it/wrme/.

We collected 5844 available data series from the time window of January 1, 1999, to December 31, 2014, from the Borkena watershed. Similarly, for UTRB, 7670 data series were collected from January 1, 1958, to December 31, 1978. Both case study datasets are multivariate and multisite. Even though we are highly concerned and chose the series of time windows with minimum data gap for both stations, the datasets contain many missing values due to different reasons. Thus, our first task was to fill the missing values with the Monte Carlo approach for this research.

The study applied linear correlation statistics to measure the strength of dependency between different input variables [25]. Even though Mehr and Gandomi [26] stated that linear correlation might mislead or provide abundant inputs, our study does no't have a huge feature size that requires intensive feature selection criteria. Hence, we adopted a linear correlation coefficient. Moreover, Kun et al. [27] concluded that Pearson correlation coefficient (PCC) is the most applicable for multiple linear regressions (MLRs), and Oyebode [28] also stated that inputs selected with PCC showed superior model accuracy. Hence, this study applied Pearson linear correlation coefficient [29, 30]. It has a value ranging between “+1” and “-1,” where “+1” indicates a positive linear correlation, “0” is no linear correlation, and “-1” shows a negative linear correlation [25]. Equation (1) calculates the Pearson correlation coefficient, and Tables 1 and 2 present the result. Correlation values between positive (0 and 0.3) and negative (0 and −0.3) show a weak linear relationship among variables [31]. However, since we have a small number of variables and data size, for this study, we decided to omit Borkena station (*T*_max_) values, which have *r* values ranging between (−0.129) and (+0.107), and the details are presented in Table 1.

(1) $$\begin{array}{c} r=\;\frac{N\sum XY-\left(\sum X\sum Y\right)}{\sqrt{\left[N\sum x^{2}-{\left(\sum X\right)}^{2}\right]\left[N\sum Y^{2}-{\left(\sum Y\right)}^{2}\right]}}. \end{array}$$

**Figure 2:**
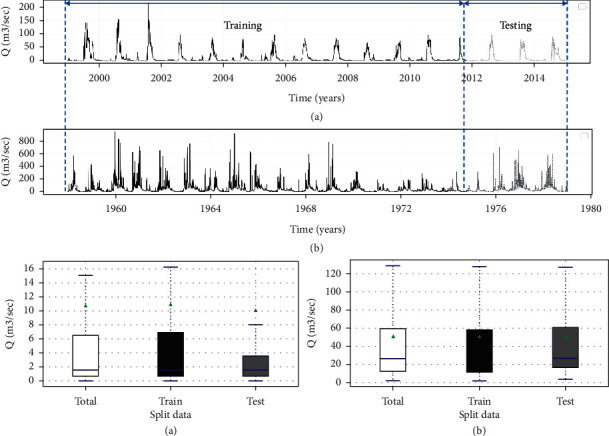
Streamflow time series graph and the corresponding box plot of split data. (a) Borkena. (b) UTRB.

After passing rigorous quality control processes, the raw data were then split chronologically into training and testing datasets with a ratio of 80 : 20, respectively. The time series graph and the corresponding box plot of split data for both stations are presented in Figure 2. Different options existed in the literature to remove noise from the time series. A sliding window is the first option to temporarily approximate the time series data's actual value [32]. In comparison, rolling windows (moving average) is the second option that smooths the time series data by calculating the average, maximum, minimum, or sum over a specific time [33]. Hence, for this study, we applied average rolling windows to smooth and remove noise from the time series by keeping the length of the data still.

Then, daily, weekly, and monthly average rolling sliding windows were used to rebuild the input and output time series into a supervised learning format. Accordingly, the rolled time series data were then prepared with the time lag window of 30 or 45 for single-step streamflow simulation at Borkena and UTRB stations, respectively. Moreover, split time series data variable scaling was performed using Standard Scaler for the modelling process's computational easiness and numerical stability.

## 4. Methods

**Figure 3:**
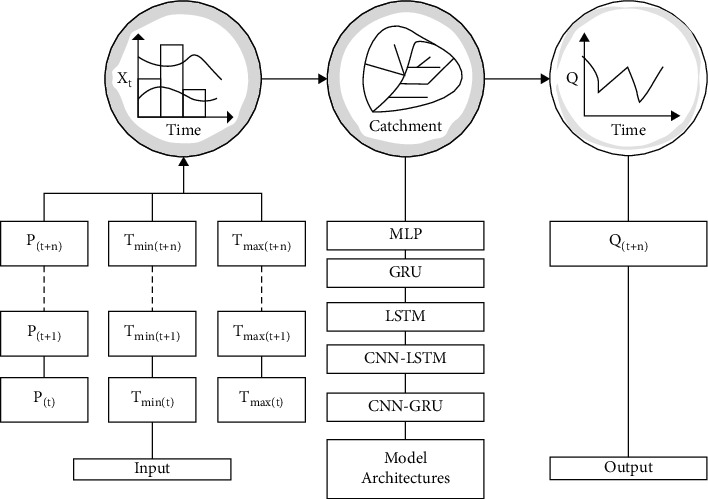
A simple architecture of the proposed models.

In this study, three types of network architectures MLP, GRU, and LSTM, were compared with the proposed hybrid deep neural network architectures CNN-LSTM and CNN-GRU for the simulation of single-step streamflow by taking different combinations of precipitation (*P*), minimum temperature (*T*_min_), and maximum temperature (*T*_max_) as inputs. The proposed simulation model architectures with their input and output variables are briefly presented as a flowchart in Figure 3.

### 4.1. Deep Learning Models

Deep learning models are part of a broader family of machine learning, including recurrent neural networks (RNNs), convolutional neural networks (CNNs), deep belief networks (DBNs), and deep neural networks (DNNs). These models have been applied to different fields of study, including speech recognition, computer vision, natural language processing, and time series analysis [13, 16, 34–36]. The following sections will briefly discuss some of these architectures that were used in the present study.

### 4.2. Artificial Neural Network (ANN)

Artificial neural network (ANN) is the most common machine learning model that has found application in streamflow simulation over the last two decades [1, 37]. It is known for modelling complex input-output relationships inherent in hydrological time series features within a river catchment. The traditional feedforward neural network (FFNN) with three layers of input-output and hidden layers trained by backpropagation (BP) algorithm gained popularity for nonlinear hydrological time series modelling.

**Figure 4:**
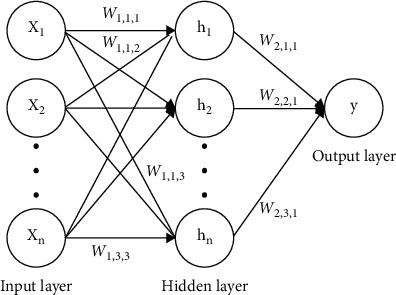
Typical architecture of ANN.

Figure 4 displays the typical architecture of ANN.

(2) $$\begin{array}{c} \hat{y_{j}}=f_{j}\left[\sum_{h=1}^{m}w_{jh}*f_{h}\left(\sum_{i=1}^{n}w_{hi}x_{i}+w_{hb}\right)+w_{jb}\right], \end{array}$$

where *i, h, j, b*, and *w* indicate neurons of the input, hidden, output layers, bias, and applied weight of the neuron, respectively; *f*_*h*_ and *f*_*j*_ show the activation functions of the hidden layer and output layer, respectively; *x*_*i*_*, n*, and *m* represent, respectively, the input value, input neuron, and hidden neuron numbers; and *y* and $\hat{{\text{y}}_{\text{j}}}$ denote the observed and calculated target values, respectively. In the calibration phase of the model, the values of the hidden and output layers and corresponding weights could be varied and calibrated [38].

The ability of ANN to link input and output variables in complex hydrological systems without the need for prior knowledge about the nature of the process has led to a huge leap in the use of ANN models in hydrological simulations [38].

### 4.3. Long Short-Term Memory (LSTM)

**Figure 5:**
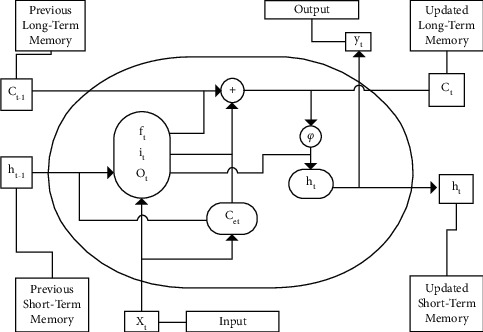
LSTM memory cell with three gated layers [11].

The difference of LSTM from the classical MLP network is that layers of the neurons in LSTM have recurrent connections; thus, the state from the previous activation time step is used to formulate an output. The LSTM replaces the typical neuron in the hidden layer with a memory cell and three gates: an input gate, a forget gate, and an output gate [39]. It is an advanced form of recurrent neural network (RNN) that can capture long-term dependencies. On the other hand, RNN is a circular network in which an additional input is added to represent the state of the neuron in the hidden layer at the previous time steps [40]. LSTM has two critical benefits over RNN: overcoming vanishing and exploding gradients and holding memory to capture long-term temporal dependency in input sequences. The mathematical formulation for different parameters is listed in Table 3, and Figure 5 displays the LSTM memory cell with three gated layers.^*∗*^*W*_*i*_, *W*_*f*_, *W*_*o*_, and *W*_*c*_ are the weights that map the hidden layer input to the three gates of input, forget, and output. *U*_*i*_*, U*_*f*_*, Uo,* and *U*_*c*_ weight matrices map the hidden layer output to gates; *bi, b*_*f*_, *b*_*o*_, and *b*_*c*_ are vectors. *C*_*t*_ and *h*_*t*_ are the outcome of the cell and the outcome of the layer, respectively.

### 4.4. Gated Recurrent Unit (GRU)

**Figure 6:**
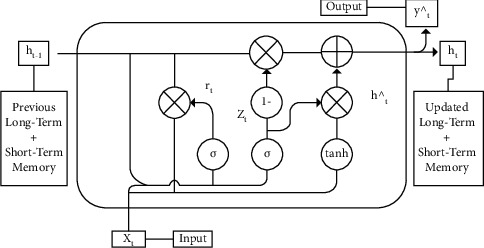
The structure of gated recurrent unit (GRU) network [34].

GRU is a special type of LSTM architecture in which it merges the input and forget gates and converts them into an update gate, which makes the parameter numbers fewer, and the training will be easier. There are two input features each time: the input vector *x*_t_ and the previous output vector *h*_t−1_. The output of each specific gate can be calculated through logical operation and nonlinear transformation of the input [34]. The mathematical formulations among inputs, outputs, and different parameters are listed in equations (3), (4), (5), and (6). Moreover, Figure 6 displays the structure of the gated recurrent unit (GRU) network.

(3) $$\begin{array}{c} Z_{t}=\sigma \left(W_{z}X_{t}+Uzh_{t-1}+b_{z}\right), \end{array}$$

(4) $$\begin{array}{c} r_{t}=\sigma \left(W_{r}X_{t}\;+U_{r}h_{t-1}+b_{r}\right), \end{array}$$

(5) $$\begin{array}{c} {\overset{⌢}{h}}_{t}=\tanh\left(W_{h}X_{t}\;+\left(r_{t}*h_{t-1}\right)+b_{h}\right), \end{array}$$

(6) $$\begin{array}{c} h_{t}=\left(1-Z_{t}\right)*h_{t-1}+Z_{t}*{\overset{⌢}{h}}_{t}, \end{array}$$

where *Z*_*t*_ is the update gate vector, *r*_*t*_ is the reset gate vector, *W* and *U* are parameter matrices, *σ* is a sigmoid function, and tanh is a hyperbolic tangent.

### 4.5. Convolutional Neural Network (CNN)

Convolutional neural network (CNN) is one of the most successful deep learning models, especially for feature extraction, and its network structures include 1D CNN, 2D CNN, and 3D CNN [15]. CNN structure generally consists of a convolution layer, a pooling layer, and a full connection layer [18].

**Figure 7:**
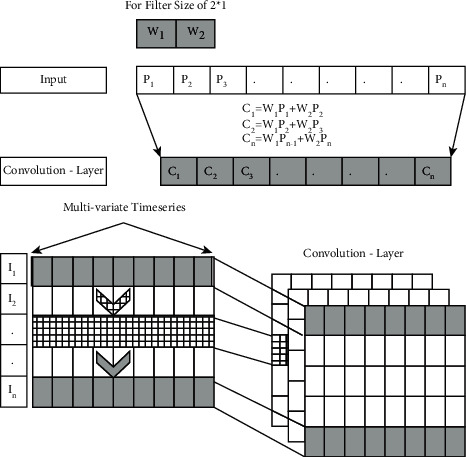
The process of 1D CNN [15].

1D CNN is mainly implemented for sequence data processing [41], 2D CNN is usually used for text and image identification [42], and usually, 3D CNN is recognized for modelling medical image and video data identification [43]. Hence, since the aim of the present study is time series analysis, we implemented 1D CNN. The detailed process of 1D CNN is described in Figure 7.

As depicted in Figure 7, the input series is convoluted to the convolution layer from top to bottom (shown by the arrows). The grey or the mesh colours represent different filters where the size of the convolution layer depends on the number of input data dimensions, the size of the filter, and the convolution step length.

### 4.6. CNN-LSTM and CNN-GRU Hybrid Models

In this study, hybrid models were designed by integrating CNN with LSTM or GRU layers. Hence, the feature sequence from the CNN layer was considered as the input for the LSTM or GRU layer, and then the short and long-time dependencies were further extracted.

**Figure 8:**
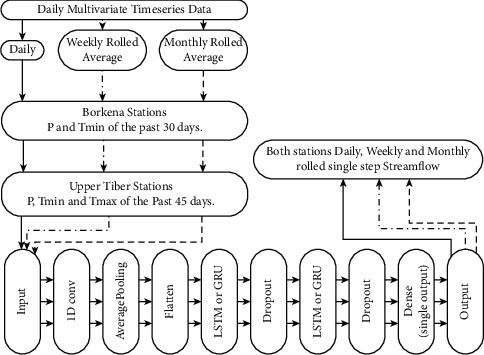
The basic architecture of the proposed CNN-LSTM or CNN-GRU models.

The proposed CNN-LSTM or CNN-GRU models contain two main components: the first component consists of one dimensional single or double convolutional and average pooling layers. Moreover, a flatten layer is connected to further process the data into the format required by the LSTM or GRU. In the second component, the generated features are processed using LSTM, GRU, and dense layers. Additionally, dropouts are introduced to prevent overfitting.  Figure 8 shows the designed model inputs and outputs with a basic description of the convolutional, pooling, and LSTM or GRU layers proposed for this project.

## 5. Data Analysis

Simulation with deep learning requires selecting a probable combination of hyperparameters: batch size, epochs, number of layers, and number of units for each layer [8]. Optimizing hyperparameters is not always consistent as there is no hard rule to follow. “The process is more of an art than a science” [44]. Hence, in this study, we chose the Keras tuner optimizer developed by the Google team and included it in the Keras open library [45, 46].

### 5.1. Hyperparameter Optimization

Tuning machine learning model hyperparameters is critical. Varying hyperparameter values often results in models with significantly different performances [47]. The models applied in this study mainly contain two types of hyperparameters: constant hyperparameters that are not altered through the optimization process and variable hyperparameters. Adam optimizer is applied under the category of the constant hyperparameters because of its efficiency and easiness to implementation that requires minimum memory and is commonly suited in different problems [48]. In this category, rectified linear unit (Relu) was used as an activation function, and mean squared error (MSE) was used as a loss function.

In contrast, the second type of changing hyperparameters is optimized by Keras tuner, and hyperparameter choices or value ranges for optimization are set using different trials. We also considered our PC capacity (processor: Intel(R) Core (TM) i7-6500U CPU 2.50 GHz and RAM: 8 gigabytes) with Windows 10 operating system. Hyperparameters are optimized with 20 trials, and since deep learning networks have different training and validation plots for each run, we decided to repeat the iteration three times.

All hyperparameter ranges or choices are listed in Table 4. CNN-LSTM_1_ and CNN-GRU_1_ models used hyperparameter values from numbers 1 to 13 for optimization (Table 4), while we omitted 4, 5, and 6 for CNN-LSTM_2_ and CNN-GRU_2_. The remaining deep learning models MLP, LSTM, and GRU used a list of hyperparameters from numbers 7 to 13. Finally, each optimized hyperparameter is used for each training and testing experiment. Moreover, the train and test traces from each run can be plotted to give a more robust idea of the behaviour of the model to inspect overfitting and underfitting issues.

### 5.2. Performance Measures

A wide variety of evaluation metrics are listed in the literature [49]. However, the popular ones are mean error (ME), coefficient of determination (*R*^2^), root mean square error (RMSE), mean absolute error (MAE), mean percentage error (MPE), mean absolute percentage error (MAPE), mean absolute scaled error (MASE), and Nash–Sutcliffe efficiency (NSE). This study used different input and model variability conditions. Hence, to concisely measure the analysis output and present the result, we applied the following top three standard performance evaluation criteria that can also have the potential to capture the extreme streamflow time series values effectively [50].

Coefficient of determination (*R*^2^):

(7) $$\begin{array}{c} R^{2}=\frac{n\left(\sum^{}Q_{obs}*Q_{sim}\right)-\left(\sum^{}Q_{obs}\right)*\left(\sum^{}Q_{sim}\right)}{\sqrt[{\left(1/2\right)}]{\left[n\left(\sum^{}Q_{obs}^{2}\right)-{\left(\sum^{}Q_{obs}\right)}^{2}\right]}*\left[n\left(\sum^{}Q_{sim}^{2}\right)-{\left(\sum^{}Q_{sim}\right)}^{2}\right]}. \end{array}$$

Root mean square error (RMSE):

(8) $$\begin{array}{c} RMSE=\;\sqrt{\frac{\sum_{n=1}^{N}{\left(Q_{0bs}^{t}-Q_{sim}^{t}\right)}^{2}}{N}}. \end{array}$$

Mean absolute error (MAE):

(9) $$\begin{array}{c} MAE=\frac{1}{n}\sum_{i=1}^{n}\left|Q_{0bs}^{t}-Q_{sim}^{t}\right|, \end{array}$$

where *Q*_*obs*_ = discharge observed, *Q*_*sim*_ = discharge simulated, and *n* = number of observations. The range of *R*^*2*^ lies between 0 and 1, representing, respectively, no correlation and a perfect correlation between observed and simulated values, whereas smallest RMSE and MAE scores or values close to zero direct to the best model performance.

## 6. Results

Streamflow simulation result with the proposed seven deep learning architectures, different input time window series, two climatic regions, two input combinations, and three average rolling time windows is presented in Tables 5, 6, and 7. Regardless of the combination of these conditions, the CNN-GRU model showed promising results in most of these scenarios (Tables 5 and 7). The highest scores are presented here.

1. In daily streamflow simulation for Borkena station, CNN-GRU_1_ scored 7.94, 3.66, 0.85, and 0.63, and for UTRB station, CNN-GRU_2_ scored 45.61, 21.79, 0.57, and 0.64 for RMSE, MAE, *R*^2^, and training time per epoch, respectively.
2. In weekly rolled streamflow simulation for Borkena station, CNN-LSTM^2^ scored 7.33, 3.86, 0.87, and 0.52, and for UTRB station, GRU scored 25.21, 14.83, 0.77, and 5.56 for RMSE, MAE, *R*^2^, and training time per epoch, respectively.
3. In monthly rolled streamflow simulation, the CNN-GRU_2_ model showed high performance with 5.15, 3.18, 0.92, and 0.78 scores for Borkena station and 17.98, 12.99, 0.83, and 0.71 for UTRB station, which are RMSE, MAE, *R*^2^, and training time per epoch, respectively.

Moreover, from the proposed four hybrid models, CNN-GRU_2_ or the model designed by a single 1D CNN layer showed the highest promising result on trial model 1(UTRB) and model 3, as shown in Tables 5 and 7. In contrast, GRU on model 2 (UTRB), CNN-LSTM_2_ on model 2 (Borkena), and CNN-GRU_1_ on model 1 (Borkena) shared the second-highest promising result. Streamflow simulation with the CNN-GRU_2_ model generally showed the highest performance than the other tested hybrid deep learning models and state-of-the-art LSTM, GRU, and MLP models. In line with our objectives, the result is discussed with different variability conditions in the following paragraphs.

### 6.1. Climatic Region Variability

Testing models in different climatic conditions with historical data will likely provide robust deep learning models for streamflow simulation in the future [51]. Hence, this research also tested different models in two climatic regions, and irrespective of climatic and time window variation, the CNN-GRU model displayed the highest scores on tested case study areas.

### 6.2. Input Combination Variability

Input combination, minimum temperature (Tmin) with precipitation (P), does not show significant performance increment in the Borkena station (Tables 5 and 6). In some scenarios, adopting P only as input increases the performance of the model (Table 7). In contrast, for UTRB, streamflow simulation with all input variables or Tmin, Tmax, and P showed significant performance increments (Table 7).

### 6.3. Average Rolling Time Window Variability

Streamflow simulation without rolling daily time series data had deficient performance compared to monthly rolled average time series. This could be because the time series noise in UTRB is visible compared to that in Borkena station. As a result, performance increment from daily to monthly rolled window models is much higher in UTRB than in Borkena station.

**Figure 9:**
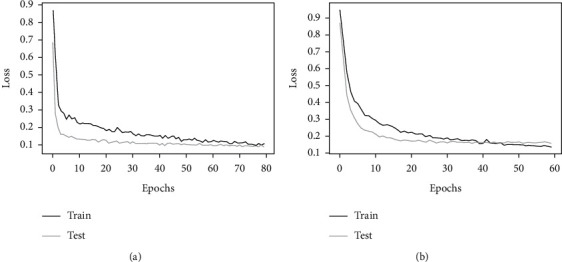
Training and test loss function of the optimized high score hybrid model. (a) CNN-GRU2 model for Borkena Station. (b) CNN-GRU2 model for UTRB Station.

**Figure 10:**
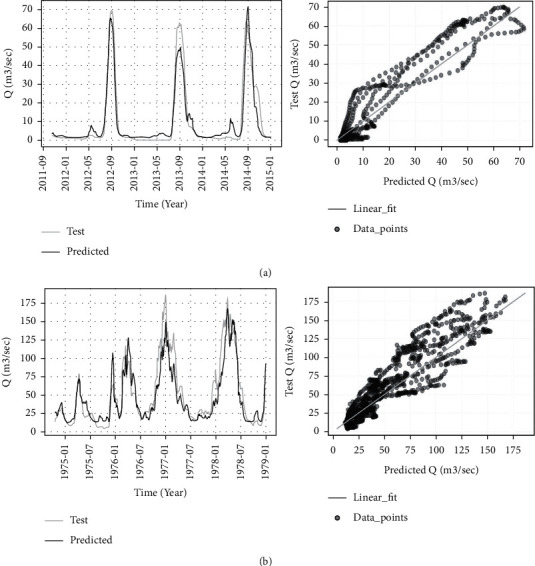
Comparison of true values and predicted values of the optimized high score hybrid model. (a) CNN-GRU_2_ model for Borkena Station. (b) CNN-GRU_2_ model for UTRB Station.

**Figure 11:**
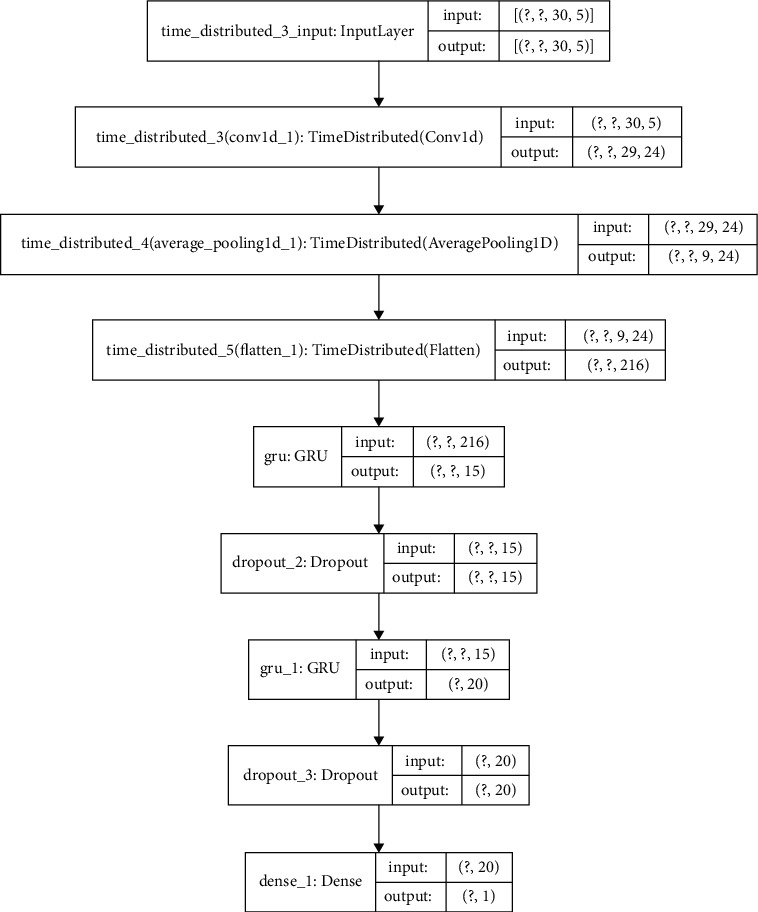
Internal network structure of the optimized high score hybrid CNN-GRU2 model for Borkena Station.

**Figure 12:**
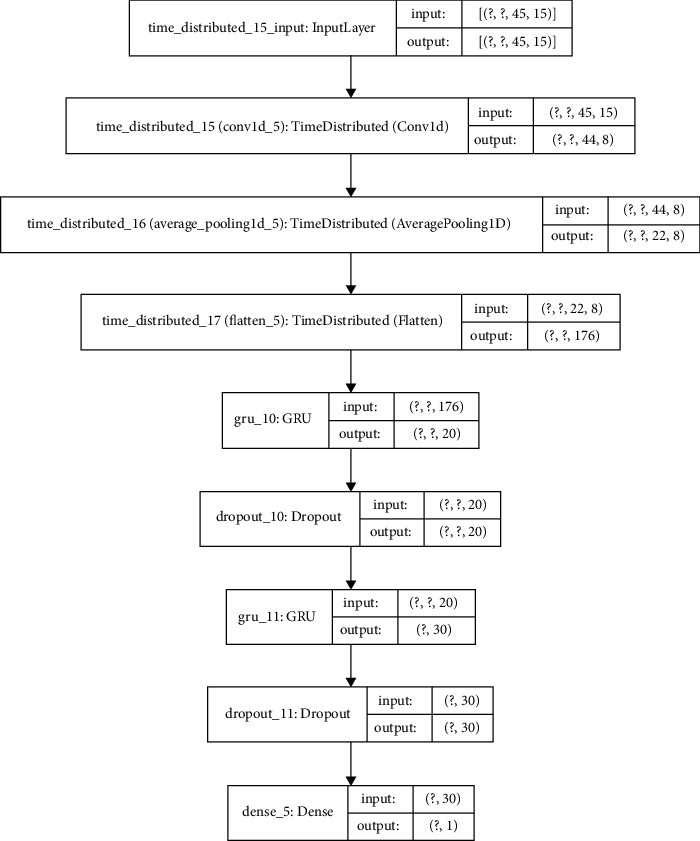
Internal network structure of the optimized high score hybrid CNN-GRU2 model for UTRB Station.

Generally, the monthly rolled time window with the CNN-GRU2 model showed the top performance results in both stations (Table 7). The corresponding training and test loss functions of this optimized high score hybrid model for both stations are displayed in Figure 9. Consequently, Figure 10 compares the true values and predicted values of this model. The optimized hybrid model boosts the performance score and lowers the training time per epoch much better than GRU and LSTM models. This model, input feature, and Keras tuner optimized hyperparameter values for both stations with its MSE score are presented in Tables 8 and 9. Moreover, the internal network structures of these models are also shown in Figures 11 and 12, which display the model input and output parameter matrices for each layer.

## 7. Conclusions

This study showed a comparative analysis of different hybrid deep learning algorithms with state-of-the-art machine learning models for one-step daily streamflow simulation at two river basins or subcatchment stream flow outlets. The proposed algorithms for this study are CNN-LSTM and CNN-GRU hybrid deep learning models, each model having one or two 1D CNN layers with the classic MLP, LSTM, and GRU models. This study conducted a series of experiments to observe the performance variation of the proposed models by introducing different input combinations, rolling time windows, and climatic conditions for streamflow simulation. The following list of points will summarize the significant findings of this study.

- CNN-GRU_2_ with one 1D CNN layer showed the best simulation performance reporting the lowest RMSE, MAE, and *R*^2^ out of all models in both case study areas. Such results dictate that the performance of the selected architectures is irrespective of the climatic characteristics of the basins.
- Combining temperature data with precipitation as input and inserting to the proposed models had minimum performance increment in Borkena station compared to UTRB case study area, which clearly showed that temperature data scarcity has more performance loss implication in UTRB station. On the other hand, the Borkena station has significant natural streamflow variability than UTRB, which is also reflected in the model results. This implies the consideration of catchment response before any deep learning model applications.
- Rolling the time window of input and output time series for streamflow simulation using the proposed models considerably increases performance in the UTRB than in the Borkena station.
- The analysis results also showed that training time per epoch for the hybrid deep learning models is much lower than that of GRU and LSTM models.

Deep learning models usually require massive datasets, and their performance drops with small to medium datasets. However, from this case study, acceptable results and considering hybrid models' hyperparameters sensitivity and complexity, future research may further design optimized configurations. Moreover, they can test these hybrid models for long-term streamflow simulation in ephemeral, seasonal, and perennial river systems and other fields of study. Our future research will try to synchronize the highly performed hybrid deep learning models in this study with remote sensing datasets for the problem we experience in the ungauged catchments.

## Figures and Tables

**Table 1:** Descriptive statistics of split time series data for the Borkena watershed.

Stations	Data type	Pearson correlation with streamflow	Training data (80%)				Testing data (20%)			
			Mean	Max	Min	SD	Mean	Max	Min	SD
Kombolcha	Stream flow (m^3^/sec)	1.000	10.9	216.9	0.00	23.2	10.1	94.8	0.0	20.2
	P (mm/day)	0.321	3.1	73.2	0.0	7.5	2.9	60.4	0.0	7.2
	*T*_min_ (^o^c)	0.271	12.5	20.9	1.5	3.3	12.5	20.6	2.6	3.4
	T_max_ (°c)	−0.099	27.2	33.6	16.4	2.5	27.3	33.0	19.6	2.1

Chefa	P (mm/day)	0.344	3.5	81.6	0.0	8.6	3.4	64.3	0.0	8.1
	*T*_min_ (^o^c)	0.266	13.3	21.5	0.1	3.7	14.1	22.2	3.9	3.5
	Tmax (oc)	−0.069	29.9	38.0	18.5	2.8	30.3	38.0	22.2	2.5

Dessie	P (mm/day)	0.335	3.5	80.6	0.0	8.6	2.9	67.0	0.0	7.3
	*T*_min_ (^o^c)	0.319	8.5	15.5	0.1	2.5	7.8	15.5	0.0	3.1
	Tmax (oc)	0.107	23.8	30.0	16.0	1.9	24.1	30.0	15.0	2.1

Kemise	P (mm/day)	0.372	3.1	81.9	0.0	8.3	2.9	72.1	0.0	7.5
	*T*_min_ (^o^c)	0.282	13.8	22.0	3.0	3.4	13.5	20.1	4.5	3.6
	Tmax (oc)	−0.129	31.0	38.3	14.0	2.7	31.9	37.8	23.5	2.4

Majete	P (mm/day)	0.347	3.3	80.7	0.0	8.6	3.3	81.3	0.0	8.6
	*T*_min_ (^o^c)	0.202	14.7	23.0	1.4	2.9	14.6	21.5	6.7	2.9
	Tmax (oc)	−0.057	28.6	37.8	17.2	2.8	29.1	38.0	20.8	2.4

**Table 2:** Descriptive statistics of split time series data for the UTRB.

Stations	Data type	Pearson correlation with streamflow	Training data (80%)				Testing data (20%)			
			Mean	Max	Min	SD	Mean	Max	Min	SD
Ponte Nuovo	Streamflow (m^3^/sec)	1.000	50.6	939.0	1.9	75.5	50.6	737.0	3.7	68.6

Castel Rigone	P (mm/day)	0.384	2.6	72.8	0.0	6.6	2.7	67.7	0.0	6.9

Montecoronaro	P (mm/day)	0.339	3.9	229.0	0.0	10.7	4.0	110.0	0.0	10.5

Perugia (ISA)	P (mm/day)	0.379	2.4	120.4	0.0	6.6	2.5	61.8	0.0	6.3
	T_min_ (^o^c)	−0.353	9.7	30.4	−9.0	6.3	9.3	25.2	−5.0	5.6
	T_max_ (^o^c)	−0.379	17.4	37.4	−4.5	8.1	16.3	33.0	0.6	7.2

Petrelle	P (mm/day)	0.345	2.51	90.0	0.0	6.9	2.7	117.1	0.0	7.4

Pietralunga	P (mm/day)	0.428	3.22	150.0	0.0	8.1	3.1	73.1	0.0	7.3

Spoleto	P (mm/day)	0.412	2.9	113.6	0.0	7.9	2.9	94.2	0.0	7.8
	T_min_ (^o^c)	−0.265	7.5	23.0	−12.6	6.4	8.8	21.7	−5.4	5.8
	T_max_ (^o^c)	−0.383	18.8	38.7	−3.5	8.6	18.7	36.8	2.0	7.8

Torgiano	P (mm/day)	0.364	2.4	141.2	0.0	7.1	2.5	62.0	0.0	6.9

Gubbio	T_min_ (^o^c)	−0.315	8.7	26.0	−12.0	5.9	6.1	19.3	−11.3	5.4
	T_max_ (^o^c)	−0.377	18.1	39.0	−8.0	8.1	17.4	34.1	−0.9	7.5

Assisi	T_min_ (^o^c)	−0.325	9.2	25.6	−11.6	6.2	8.2	21.5	−8.0	5.6
	T_max_ (^o^c)	−0.378	18.2	37.8	−5.0	8.3	18.1	35.8	0.0	7.8

**Table 3:** Mathematical formulation for LSTM cell.

Network gates	Purpose	Equations^*∗*^
Forget gate	Chooses the information to reject from the cell	*f*_*t*_ *=σ (u*_*f*_ *x*_*t*_ *+* *w*_*f*_*h*_*t-1*_*+ b*_*f*_)
Input gate	Decides what information is relevant to update in the current cell state	*i*_*t*_ *=σ (u*_*i*_ *x*_*t*_ *+* *w*_*i*_*h*_*t-1*_*+ b*_*i*_)
Output gate	Decides what to output based on input and the long-term memory of the cell	*o*_*t*_ *=σ (u*_*o*_ *x*_*t*_ *+* *w*_*o*_*h*_*t-1*_*+ b*_*o*_)
Cell state	Long-term memory	*C*_*et*_ *=* tanh *(W*_*c*_*X*_*t*_ *+* *U*_*c*_*h*_*t−1*_*+ b*_*c*_)
		*C*_*t*_ *=f*_*t*_ *∗ C*_*t−1*_ * + i*_*t*_ * ∗ C*_*et*_
Hidden state	Short-term memory	*h*_*t*_ *=* tanh *(C*_*t*_*) ∗ O*_*t*_

**Table 4:** Model hyperparameter choices or value ranges for optimization by Keras tuner.

N^o^	Hyperparameters	Value ranges^*∗∗*^			Choices	Default
		Min	Max	Step		
1	Conv_1_filter	8	32	8	^*∗*^	^*∗*^
2	Conv_1_kernal	^*∗*^	^*∗*^	^*∗*^	2 or 3	^*∗*^
3	Conv_1_pool_size	^*∗*^	^*∗*^	^*∗*^	2 or 3	^*∗*^
4	Conv_2_filter	8	32	8	^*∗*^	^*∗*^
5	Conv_2_kernal	^*∗*^	^*∗*^	^*∗*^	2 or 3	^*∗*^
6	Conv_2_pool_size	^*∗*^	^*∗*^	^*∗*^	2 or 3	^*∗*^
7	CNN-LSTM_1_, CNN-LSTM_2_, CNN-GRU_1_, CNN-GRU_2_, LSTM, GRU, or MLP layer 1 units	5	30	5	^*∗*^	^*∗*^
8	Dropout 1	0.0	0.3	0.1	^*∗*^	0.2
9	CNN-LSTM_1_, CNN-LSTM_2_, CNN-GRU_1_, CNN-GRU_2_, LSTM, GRU, or MLP layer 2 units	5	30	5	^*∗*^	^*∗*^
10	Dropout 2	0.0	0.3	0.1	^*∗*^	0.2
11	Learning rate	^*∗*^	^*∗*^	^*∗*^	1e-2, 1e-3 or 1e-4	^*∗*^
12	Number of epochs	10	100	10	^*∗*^	^*∗*^
13	Number of batch sizes	10	100	10	^*∗*^	^*∗*^

^*∗∗*^Value ranges or choices for optimization by Keras tuner: (objective = “validation loss,” max trials = 20, and executions per trial = 3). ^*∗*^Not applicable.

**Table 5:** Daily streamflow simulation: performance comparison of the proposed models for different input variables and climatic conditions.

Model 1	Borkena								UTRB							
	*P* + Tmin				*P*				*P* + Tmin + Tmax				*P*			
	R	M	*R* ^2^	T	R	M	*R* ^2^	T	R	M	*R* ^2^	T	R	M	*R* ^2^	T
	M	A		T	M	A		T	M	A		T	M	A		T
	S	E		P	S	E		P	S	E		P	S	E		P
	E			E^*∗*^ (sec)	E			E^*∗*^ (sec)	E			E^*∗*^ (sec)	E			E^*∗*^ (sec)
MLP	9.91	5.01	0.77	0.89	9.38	4.63	0.79	0.63	49.11	22.74	0.49	0.78	56.57	28.14	0.33	0.41
GRU	8.78	4.37	0.82	3.61	7.94	3.64	0.85	3.32	46.63	20.89	0.55	2.61	51.09	26.74	0.45	3.39
LSTM	8.41	4.09	0.83	2.35	9.65	4.87	0.78	2.92	48.64	22.79	0.51	3.86	48.59	25.00	0.51	5.98
CNN-LSTM_1_	8.09	4.07	0.84	0.46	8.57	4.67	0.82	0.41	51.20	22.95	0.45	1.19	56.16	26.55	0.34	0.57
CNN-LSTM_2_	7.99	4.09	0.85	0.72	9.14	4.50	0.80	0.45	45.38	21.85	0.57	0.82	51.57	25.84	0.44	1.85
CNN-GRU_1_	**7.94**	**3.66**	**0.85**	**0.63**	8.32	4.09	0.83	0.86	55.06	23.49	0.37	1.16	52.42	24.98	0.43	0.83
CNN-GRU_2_	9.07	4.19	0.80	1.01	8.43	4.26	0.83	0.28	**45.61**	**21.79**	**0.57**	**0.64**	49.96	25.38	0.48	0.68

^*∗*^TTPE (training time per epoch). The bold values indicate the highest performance score.

**Table 6:** Weekly rolled streamflow simulation: performance comparison of the proposed models for different input variables and climatic conditions.

Model 2	Borkena								UTRB							
	*P* + Tmin				*P*				*P* + Tmin + Tmax				*P*			
	R	M	*R* ^2^	T	R	M	*R* ^2^	T	R	M	*R* ^2^	T	R	M	*R* ^2^	T
	M	A		T	M	A		T	M	A		T	M	A		T
	S	E		P	S	E		P	S	E		P	S	E		P
	E			E^*∗*^ (sec)	E			E^*∗*^ (sec)	E			E^*∗*^ (sec)	E			E^*∗*^ (sec)
MLP	8.11	4.29	0.84	0.23	7.33	4.19	0.87	0.22	33.01	20.01	0.60	0.74	38.03	25.17	0.47	0.79
GRU	7.59	3.71	0.86	2.04	7.15	4.13	0.87	2.43	**25.21**	**14.83**	**0.77**	**5.56**	31.39	19.26	0.64	16.79
LSTM	8.41	4.01	0.82	2.98	7.93	3.91	0.84	1.27	31.07	18.87	0.65	3.55	31.07	19.49	0.65	2.69
CNN-LSTM_1_	7.90	4.09	0.85	0.78	7.72	4.25	0.85	0.63	28.04	17.33	0.71	0.93	34.57	21.92	0.57	0.62
CNN-LSTM_2_	**7.33**	**3.86**	**0.87**	**0.52**	7.63	4.25	0.86	0.55	28.45	16.66	0.71	1.14	35.04	21.77	0.55	1.56
CNN-GRU_1_	7.83	3.94	0.85	0.44	7.91	4.31	0.85	0.50	30.57	18.01	0.66	2.32	35.14	22.58	0.55	0.63
CNN-GRU_2_	8.73	4.61	0.81	0.43	8.43	4.35	0.82	0.97	27.81	16.99	0.72	4.37	33.76	23.01	0.59	1.01

^*∗*^TTPE (training time per epoch). The bold values indicate the highest performance score.

**Table 7:** Monthly rolled streamflow simulation: performance comparison of the proposed models for different input variables and climatic conditions.

Model 2	Borkena								UTRB							
	*P* + Tmin				*P*				*P* + Tmin + Tmax				*P*			
	R	M	*R* ^2^	T	R	M	*R* ^2^	T	R	M	*R* ^2^	T	R	M	*R* ^2^	T
	M	A		T	M	A		T	M	A		T	M	A		T
	S	E		P	S	E		P	S	E		P	S	E		P
	E			E^*∗*^ (sec)	E			E^*∗*^ (sec)	E			E^*∗*^ (sec)	E			E^*∗*^ (sec)
MLP	6.68	4.37	0.87	0.58	5.57	3.80	0.91	0.41	20.24	13.84	0.78	0.44	28.79	21.05	0.56	0.41
GRU	5.15	3.52	0.91	1.62	5.22	3.06	0.92	3.31	20.79	14.30	0.77	16.63	26.47	20.08	0.63	4.70
LSTM	5.55	3.49	0.91	2.75	5.76	3.51	0.90	2.51	21.49	15.11	0.76	4.15	32.29	24.47	0.45	5.09
CNN-LSTM_1_	6.05	4.42	0.89	0.98	5.58	3.40	0.91	0.58	21.53	14.87	0.76	1.29	27.48	21.19	0.60	0.42
CNN-LSTM_2_	5.36	3.17	0.92	1.41	6.87	4.05	0.86	1.44	19.07	13.53	0.81	0.70	27.79	20.90	0.59	0.42
CNN-GRU_1_	5.76	3.62	0.90	0.52	5.77	3.56	0.90	0.69	19.31	13.78	0.80	4.87	28.67	21.07	0.57	3.08
CNN-GRU_2_	5.36	3.25	0.92	0.62	**5.15**	**3.18**	**0.92**	**0.78**	**17.98**	**12.99**	**0.83**	**0.71**	27.77	20.36	0.59	1.22

^*∗*^TTPE (training time per epoch). The bold values indicate the highest performance score.

**Table 8:** Best hybrid model type, input feature, and Keras tuner optimized hyperparameter values for Borkena station with its MSE score.

Hyperparameters:	CNN-GRU_2_
	Monthly rolled P
Conv_1_filter	24
Conv_1_kernal	2
Conv_1_pool_size	3
GRU_l1_units	15
Dropout1	0.1
GRU_l2_units	20
Dropout2	0.2
Learning rate	0.0001
Number of epochs	80
Number of batch sizes	20
Score (MSE)	0.083

**Table 9:** Best hybrid model type, input features, and Keras tuner optimized hyperparameter values for UTRB station with its MSE score.

Hyperparameters:	CNN-GRU_2_
	Monthly rolled P, *T*_min_ and *T*_max_
Conv_1_filter	8
Conv_1_kernal	2
Conv_1_pool_size	2
GRU_l1_units	20
Dropout1	0.3
GRU_l2_units	30
Dropout2	0.2
Learning rate	0.0001
Number of epochs	60
Number of batch sizes	40
Score (MSE)	0.193

## Data Availability

The raw hydrological and metrological datasets used for the Borkena watershed are available from the corresponding author upon request. However, authorization letters are required from the Ministry of Water Irrigation and Energy (MoWIE) of Ethiopia (http://mowie.gov.et/) and the National Meteorological Agency of Ethiopia (NMA) (http://www.ethiomet.gov.et), whereas for UTRB, the datasets can be retrieved from an online repository (http://hydrogate.unipg.it/wrme/).
